# Cross-Frequency Coupling in Descending Motor Pathways: Theory and Simulation

**DOI:** 10.3389/fnsys.2019.00086

**Published:** 2020-01-14

**Authors:** Nirvik Sinha, Julius P. A. Dewald, Charles J. Heckman, Yuan Yang

**Affiliations:** ^1^Department of Physical Therapy and Human Movement Sciences, Feinberg School of Medicine, Northwestern University, Chicago, IL, United States; ^2^School of Medical Science and Technology, Indian Institute of Technology, Kharagpur, India; ^3^Department of Biomedical Engineering, Robert R. McCormick School of Engineering and Applied Science, Northwestern University, Evanston, IL, United States; ^4^Department of Physiology, Feinberg School of Medicine, Northwestern University, Chicago, IL, United States

**Keywords:** cross-frequency coupling, descending motor pathways, computer simulation, Hogdkin–Huxley styled neuron model, n:m coherence

## Abstract

Coupling of neural oscillations is essential for the transmission of cortical motor commands to motoneuron pools through direct and indirect descending motor pathways. Most studies focus on iso-frequency coupling between brain and muscle activities, i.e., cortico-muscular coherence, which is thought to reflect motor command transmission in the mono-synaptic corticospinal pathway. Compared to this direct pathway, indirect corticobulbospinal motor pathways involve multiple intermediate synaptic connections via spinal interneurons. Neuronal processing of synaptic inputs can lead to modulation of inter-spike intervals which produces cross-frequency coupling. This theoretical study aims to evaluate the effect of the number of synaptic layers in descending pathways on the expression of cross-frequency coupling between supraspinal input and the cumulative output of the motoneuron pool using a computer simulation. We simulated descending pathways as various layers of interneurons with a terminal motoneuron pool using Hogdkin–Huxley styled neuron models. Both cross- and iso-frequency coupling between the supraspinal input and the motorneuron pool output were computed using a novel generalized coherence measure, i.e., n:m coherence. We found that the iso-frequency coupling is only dominant in the mono-synaptic corticospinal tract, while the cross-frequency coupling is dominant in multi-synaptic indirect motor pathways. Furthermore, simulations incorporating both mono-synaptic direct and multi-synaptic indirect descending pathways showed that increased reliance on a multi-synaptic indirect pathway over a mono-synaptic direct pathway enhances the dominance of cross-frequency coupling between the supraspinal input and the motorneuron pool output. These results provide the theoretical basis for future human subject study quantitatively assessing motor command transmission in indirect vs. direct pathways and its changes after neurological disorders such as unilateral brain injury.

## Introduction

The human motor system is a highly cooperative network comprised of different groups of neurons. Neural coupling, i.e., the synchronization of neural activity across these groups, is key to signal transmission among functionally related, though anatomically distant, neuronal groups (e.g., the motor cortices and spinal motoneuron pool) through direct and indirect descending pathways (van Wijk et al., 2012). Over decades, most researchers investigating neural coupling in the motor descending pathways have focused on the synchronization between cortical oscillations and muscle activities at the same frequency (i.e., iso-frequency coupling), known as the cortico-muscular coherence (Mima and Hallett, 1999). It is thought to reflect motor command transmission in the mono-synaptic corticospinal tract (Schoffelen et al., 2005). Previous simulation and *in vivo* studies demonstrated that, in this direct descending pathway, despite the non-linearity of individual neurons, neural oscillation of the supraspinal input could be linearly transmitted to the cumulative output of the motoneuron pool at the same frequency (Negro and Farina, 2011a,b). These previous studies explained the origin of iso-frequency coupling between the supraspinal input and the motoneuron pool output with respect to the use of the monosynaptic corticospinal tract as the fastest, direct descending pathways in healthy individuals.

However, the corticospinal tract is not the only motor pathway in humans. There are other indirect pathways (e.g., cortico-reticulospinal tract, rubrospinal tract) in parallel with the direct corticospinal tract (Dum and Strick, 1991; Jang and Seo, 2014). Although contributions from these indirect motor pathways are relatively small compared to the corticospinal tract in healthy individuals, they do still play important roles in various motor control tasks such as postural control during movement (Drew et al., 2004). Furthermore, in some neurological disorders, such as unilateral brain injury, the reliance on these indirect motor pathways may increase due to losses of corticospinal projections (Fries et al., 1993; Jang et al., 2013; Owen et al., 2017). The injury-induced increased reliance on these indirect motor pathways is likely associated with motor impairments (e.g., abnormal limb synergies and spasticity) post unilateral brain injury (Ellis et al., 2012, 2017; McPherson et al., 2018a,c; Li et al., 2019). Thus, investigating the neural coupling in these indirect motor pathways will allow for a more complete understanding of the transmission of motor commands from the brain to muscles, and may pave the way for quantitative assessments of the usage of indirect motor pathways in both normal and pathological motor control.

Compared to the direct corticospinal tract, these indirect motor pathways involve multiple synaptic connections via interneurons. Neuronal processing of synaptic inputs can lead to the modulation of inter-spike intervals which produces cross-frequency coupling, i.e., synchronization across different frequencies between input and output (Koch and Segev, 2000; Markram, 2003; Yang et al., 2018). Our previous work on multi-synaptic ascending sensory pathways (Yang et al., 2016b; Tian et al., 2018), as well as a recent opinion article (Yang et al., 2018), argued that multi-synaptic interaction in a neural pathway can lead to a substantial expression of cross-frequency coupling. However, insights into possible mechanisms underlying neural coupling in the multi-synaptic descending motor pathways are currently lacking. Focusing on the iso-frequency coupling (e.g., cortico-muscular coherence) only one previous study indicated that the input from the indirect motor pathways can reduce the iso-frequency coupling between the cortical input and motoneuron pool output (Negro and Farina, 2011a) while no insight has been provided into the neural mechanisms of cross-frequency coupling. This study aims to systematically evaluate the effect of the number of synaptic connections or interneuron layers on the expression of cross-frequency coupling between supraspinal input and output of the motoneuron pool using computer simulations. We hypothesize that multi-synaptic interaction in an indirect descending motor pathway increases the non-linear distortion of efferent motor signal transmission, resulting in enhanced cross-frequency coupling over iso-frequency coupling.

To test our hypothesis, we simulated descending pathways as various layers of interneurons in cascade with a terminal motoneuron pool, using Hodgkin-Huxley styled neuron models (Booth et al., 1997; Rybak et al., 2006). Both cross- and iso-frequency coupling between the input (which comprised of a supraspinal drive with an independent membrane noise) and the output of the motoneuron pool were computed using a recently developed generalized coherence method (Yang et al., 2016b). The ratio of cross- to iso-frequency coupling was calculated to determine which type (cross- or iso-frequency) of neural coupling is dominant and how it changes with an increasing number of synaptic connections or interneuron layers.

## Methods

### Motoneuron and Interneuron Models

We simulated descending pathways as various layers of interneurons in a cascade with a terminal motoneuron pool. All neurons were modeled in the Hodgkin–Huxley style. A two-compartment model comprising of a soma and a dendrite was used for simulating motoneurons (Booth et al., 1997). Because of the lack of adequate experimental data, a single compartment simplification of this model was used to simulate the interneurons (Rybak et al., 2006).

The *motoneuron* model incorporated the following ionic currents (with the corresponding channel conductances): fast sodium (*I*_Na_ with maximal conductance *g*_*Na*_), persistent (slowly inactivating) sodium (I_NaP_ with maximal conductance g_NaP_), delayed-rectifier potassium (*I*_K_ with maximal conductance *g*_*K*_), calcium-N (*I*_CaN_ with maximal conductance *g*_*CaN*_), calcium-L (*I*_CaL_ with maximal conductance *g*_*CaL*_), calcium-dependent potassium (*I*_K,Ca_ with maximal conductance *g*_*K,Ca*_), and leakage (*I*_L_ with constant conductance *g*_*L*_) currents (Lee and Heckman, 2001; Darbon et al., 2004; Rybak et al., 2006; Streit et al., 2006):

$$\begin{array}{c} I_{\text{Na}}=g_{Na}\times m_{Na}^{3}\times h_{Na}\times \left(V-E_{Na}\right);\\ I_{\text{NaP}}=g_{NaP}\times m_{NaP}\times h_{NaP}\times \left(V-E_{Na}\right);\\ I_{\text{K}}=g_{K}\times m_{K}^{4}\times \left(V-E_{K}\right); \end{array}$$

$$\begin{array}{l} I_{\text{CaN}}=g_{CaN}\times m_{CaN}^{2}\times h_{CaN}\times \left(V-E_{Ca}\right);\\ I_{\text{CaL}}=g_{CaL}\times m_{CaL}\times \left(V-E_{Ca}\right);\\ I_{\text{K},\text{Ca}}=g_{K,Ca}\times m_{K,Ca}\times \left(V-E_{K}\right);\\ I_{\text{L}}=g_{L}\times \left(V-E_{L}\right); \end{array}$$

where *V* is the membrane potential of the corresponding neuron compartment [i.e., soma (*V*_(*S*)_) or dendrite (*V*_(*D*)_)] in two-compartment models, or the neuron membrane potential *V* in the one-compartment interneuron model which is explained later). *E*_*Na*_, *E*_*K*_, *E*_*Ca*_, and *E*_*L*_ are the reversal potentials for sodium, potassium, calcium and leakage currents, respectively. The variables *m* and *h* (with subscripts indicating ionic channels) represent the activation and inactivation variables of the corresponding ionic channels, as described by the following differential equations:

$$\begin{array}{l} \tau_{mi}\left(V\right)\frac{d}{dt}m_{i}=m_{\infty i}\left(V\right)-m_{i}\\ \;\tau_{hi}\left(V\right)\frac{d}{dt}h_{i}=h_{\infty i}\left(V\right)-h_{i} \end{array}$$

where *i* indicates the name of the channel, m_∞*i*_(V) and h_∞*i*_(V) represent the voltage-dependent steady-state activation and inactivation, and τ_mi_(V) and τ_hi_(V) are the corresponding time constants (see Booth et al., 1997; Rybak et al., 2006 for details of these parameters). The instantaneous value of *m*_*K,Ca*_ was calculated from the intracellular Ca^2+^ concentration of the corresponding compartment as (Booth et al., 1997):

$$\begin{array}{l} m_{K,Ca}=\;\frac{Ca}{Ca+K_{d}} \end{array}$$

where *Ca* is the Ca^2+^ concentration of the corresponding compartment of the neuron and *K*_*d*_ is the half-saturation level of this conductance. The kinetics of intracellular Ca^2+^ concentration (|Ca|) were computed separately for each compartment according to the following equation:

$$\begin{array}{l} \frac{d}{dt}|Ca|=-f\times \left(\alpha I_{Ca}+k_{Ca}|Ca|\right) \end{array}$$

where *f* defines the percentage of free to total Ca^2+^, α converts the total Ca^2+^ current, *I*_Ca_, to Ca^2+^ concentration and *k*_Ca_ represents the Ca^2+^ removal rate.

The maximal channel conductances, equilibrium potentials and membrane capacitance of the neuron models were set with the same values as in Rybak et al. (2006). The details are specified in the Appendix. The equilibrium leakage potentials of the motoneurons and interneurons were set as described in section Simulations.

The dendrite–soma coupling currents (with conductance *g*_*C*_) for soma (*I*_*C*(*S*)_) and dendrite (*I*_*C*(*D*)_) were calculated as (Booth et al., 1997):

$$\begin{array}{l} \;I_{\text{C}\left(\text{S}\right)}=\frac{g_{C}}{p}\left(V_{\left(D\right)}-V_{\left(S\right)}\right)\\ I_{\text{C}\left(\text{D}\right)}=\frac{g_{C}}{1-p}\left(V_{\left(S\right)}-V_{\left(D\right)}\right) \end{array}$$

where *p* is the parameter defining the ratio of somatic surface area to the total neuronal surface area.

We used conductance-based excitatory post-synaptic potentials (EPSPs) for simulating the synaptic inputs to each motoneuron. The synapses were modeled as exponentially decaying injected currents (*I*_*SynE*_ with peak conductance *g*_*SynE*_ and reversal potential *E*_*SynE*_): *I*_*SynE*_ = *g*_*SynE*_ × (*V* – *E*_*SynE*_) into the soma compartment (Negro and Farina, 2011a). The time constant τ_*synE*_ for the decay was 5 ms (Rybak et al., 2006). The peak conductance value for synapses on motoneurons was adjusted to produce an EPSP peak of 100 μV (Finkel and Redman, 1983).

With the inclusion of *I*_*NaP*_ to the motoneuron dendrite (Rybak et al., 2006), the membrane potentials of the motoneuron soma (*V*_(S)_) and dendrite (*V*_(D)_) were computed from the following equations:

$$\begin{array}{l} C\frac{dV_{\left(S\right)}}{dt}=-I_{Na\left(S\right)}-I_{K\left(S\right)}-I_{CaN\left(S\right)}-I_{K,Ca\left(S\right)}-I_{L\left(S\right)}\\ -I_{C\left(S\right)\;}-I_{SynE}\\ C\frac{dV_{\left(D\right)}}{dt}=-I_{NaP\left(D\right)}-I_{CaN\left(D\right)}-I_{CaL\left(D\right)}-I_{K,Ca\left(D\right)}-I_{L\left(D\right)}\\ -I_{C\left(D\right)} \end{array}$$

where *C* is the membrane capacitance and *t* is time.

The *interneurons* (single-compartment models) contain only a minimal set of ionic currents (Rybak et al., 2006):

$$\begin{array}{l} C\frac{dV}{dt}=-I_{Na}-I_{K}-I_{L}-I_{SynE} \end{array}$$

There is no existing literature reporting experimentally observed values of interneuron EPSPs of the descending pathways. Since interneurons are usually much smaller than motoneurons, they have higher input resistances and smaller somatic surface areas (Bui et al., 2003). Thus, we adjusted peak conductance of synapses on interneurons to produce an EPSP peak of 500 μV.

### Input Signal and Connection Configuration

**Figure 1:**
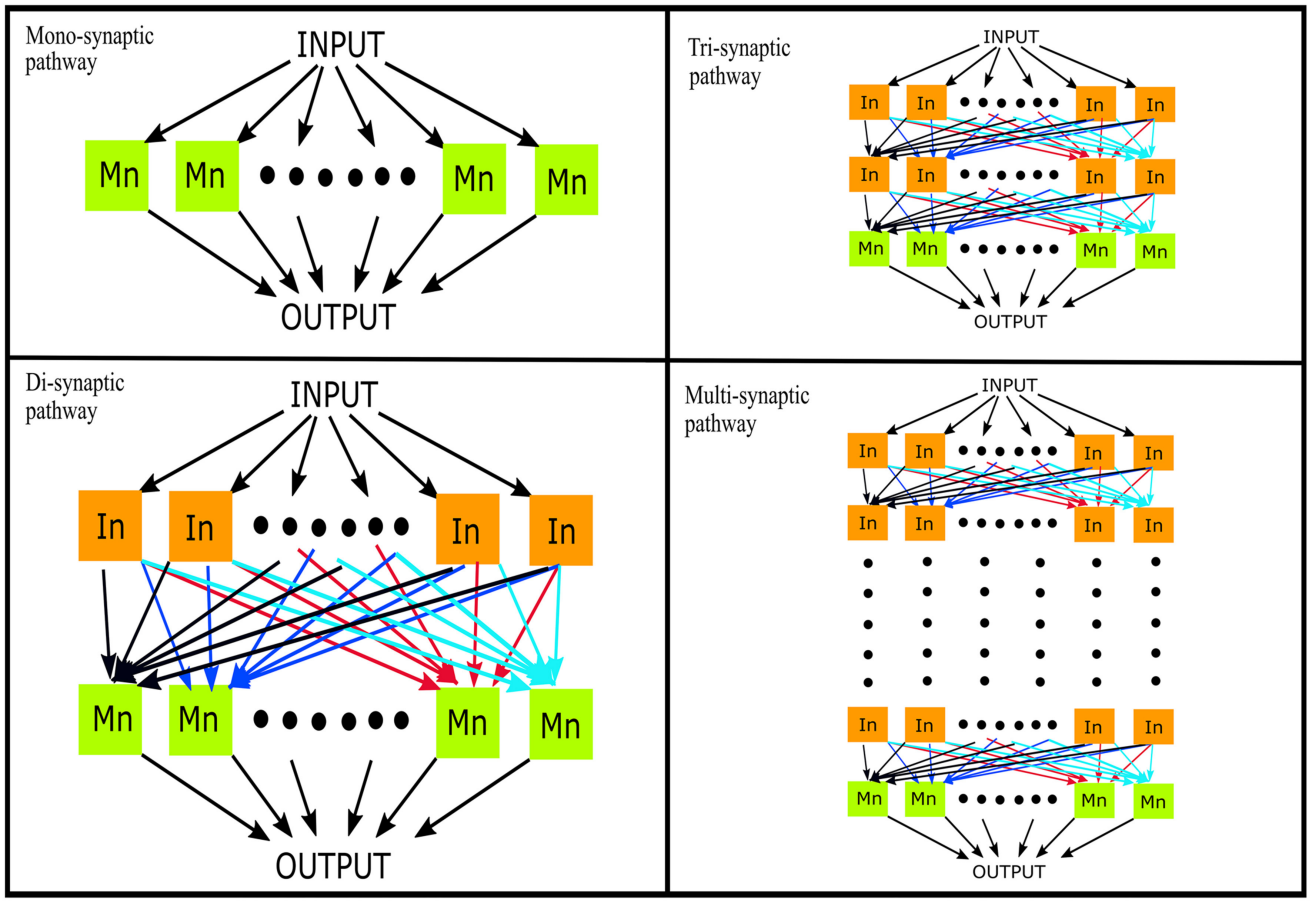
Simulation of descending pathways with various layers (*N* = 0, 1, 2, …) of interneurons in cascade with a terminal motoneuron pool. In, interneuron; Mn, motoneuron.

**Figure 2:**
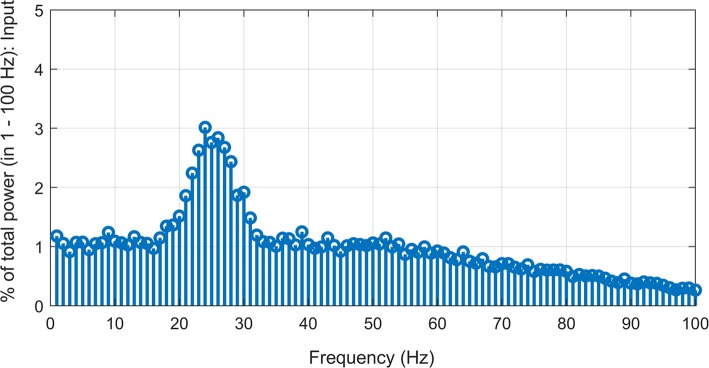
Simulated supraspinal input comprising of a Gaussian signal (15–35 Hz) with added Gaussian noise (1–100 Hz band-limited) with signal-to-noise ratio of ~−7.5 dB.

We used a probabilistic connection model (Ferrario et al., 2018) to simulate descending pathways with various layers and 100 neurons per layer (Lüscher et al., 1983) (see Figure 1). Each neuron in the first layer was fed by a time-varying injected current into the somatic compartment. The supraspinal input was designed as a Gaussian signal in beta band (15–35 Hz) [mimicking the cortical oscillations observed experimentally during motor tasks (Pfurtscheller and Da Silva, 1999)] with an added membrane noise (see Figure 2). The membrane noise was modeled as a bandlimited (1–100 Hz) Gaussian noise, which was independent for each neuron (Maltenfort et al., 1998). The total variance of this stochastic input was a percentage of the constant current injection to produce a mean ISI-CoV of (i) 0.55 for the 1st interneuron layer in case of multi-synaptic pathways (Prut and Perlmutter, 2003), and (ii) 0.2–0.3 for the motoneuron pool in case of the mono-synaptic pathway (Tanji and Kato, 1972; Sturm et al., 1997; Mattei and Schmied, 2002).

For the successive layers, the input to each neuron was the sum of output spike trains (convolved with the EPSP) of neurons randomly sampled from the previous layer. The number of neurons which contributed to the input of each interneuron was set to obtain a mean firing rate in the range of 19–24 spikes/s for the whole interneuron layer. This is in line with previous experimental observations in primate models during flexion/extension tasks (Prut and Perlmutter, 2003). The number of inputs to each motoneuron was set to 100 i.e., the sum of inputs from all interneurons of the terminal interneuron layer. The range of the firing rates was adjusted as explained in section Simulations. The mean firing rate of the active motoneurons (>8 spikes/s; Negro and Farina, 2011a) was thus obtained to be in the range of 16–19 spikes/s. Such a connection model resembles the anatomical course of various descending motor pathways which, via a varying number of interneuron layers, terminate on spinal motoneuron pools (Matsuyama et al., 2004).

### Simulations

**Figure 3:**
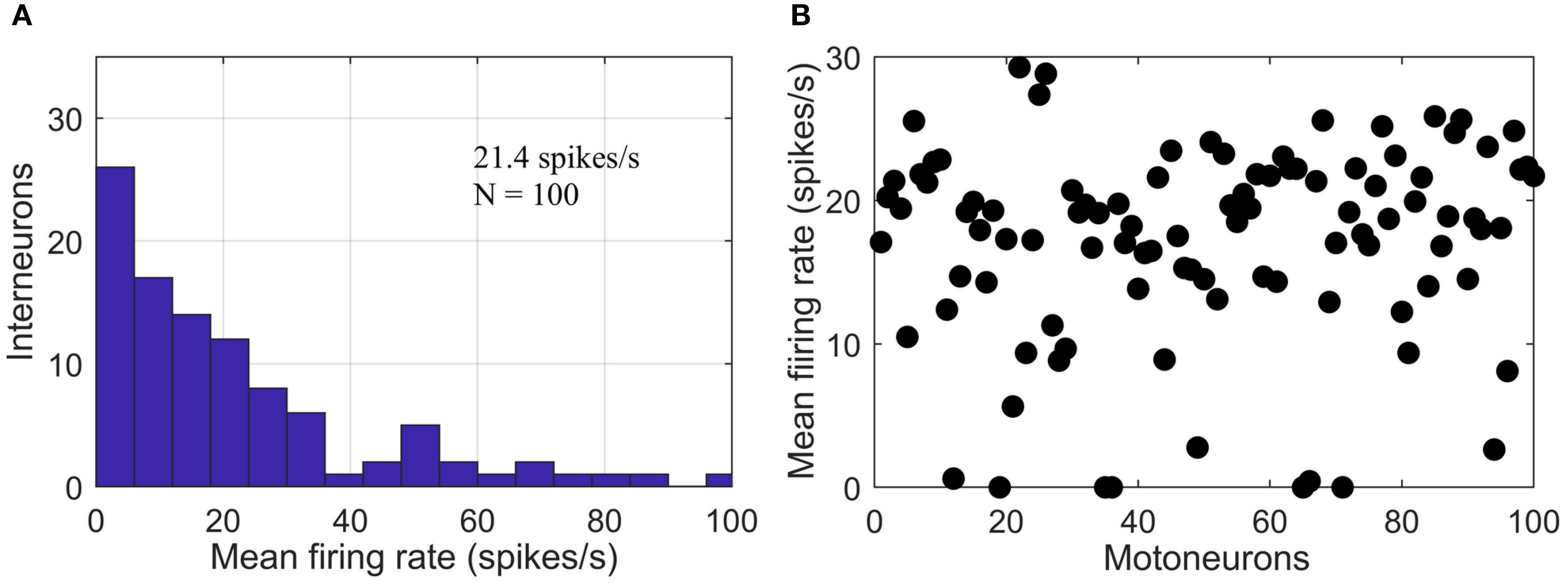
Firing rate of simulated neurons. **(A)** Histogram of mean firing rates of the simulated interneuron layer with a secondary peak at ~ 50 spikes/s. **(B)** Scatter plot showing the distribution of mean firing rates of the simulated pool of motoneuron.

Simulations were run at a sampling rate of 1 kHz using 200 epochs with a 1-s duration per epoch. The resulting data were sufficient for a robust neural coupling analysis (Hagihira et al., 2001). Our simulated multi-synaptic pathways represented the part of descending pathway involving only spinal interneurons and motoneurons, since no reticular neurons were simulated due to the complete lack necessary parameters in the existing literature (McDougal et al., 2017). A previous study reported heterogeneity of excitatory spinal interneuron populations based on their firing rates. It found a non-monotonous decline in the mean firing rate histogram with a local peak at ~50 spikes/s (Prut and Perlmutter, 2003). In the simulations, we mimicked this histogram (see Figure 3A) by combining two random exponential distributions of leakage potential (*E*_*L*_) values for each interneuron layer. The range of *E*_*L*_ was adjusted so that its mean was around −64 mV (Rybak et al., 2006). Motoneuron firing rates have been experimentally reported to be predominantly in the range of 5–30 spikes/s during isometric contractions of limb muscles (for contraction levels ≤60% of maximum voluntary torque) (De Luca and Hostage, 2010). We adjusted the leakage potentials of the motoneurons in the pool to generate a distribution (Rybak et al., 2006) of firing rates in a similar range (see Figure 3B).

### Neural Coupling Analysis

We used our recently developed generalized coherence measure, i.e., n:m coherence (NMC) (Yang et al., 2016b), to assess cross- and iso-frequency coupling between the simulated input and output signals. The n:m coherence is a straightforward extension of the linear coherence used in corticomuscular coherence (Mima and Hallett, 1999) based on high-order statistics (Nikias and Mendel, 1993) for distinguishably determining cross- and iso-frequency coupling between signals. Thus, the iso-frequency coupling of our results obtained by this method would be comparable to previous corticomuscular coherence studies (Mima and Hallett, 1999; Mima et al., 2001; Yang et al., 2016a, 2018).

Let *X*(*f*), *Y*(*f*) be the Fourier Transform of two time series (e.g., the input and output signals). The NMC between them is defined as:

$$\begin{array}{l} NMC\left(f_{X},f_{Y}\right)=\frac{\left|S_{XY}\left(f_{X},f_{Y}\right)\right|}{\sqrt{S_{X}^{n}\left(f_{X}\right)S_{Y}^{m}\left(f_{Y}\right)}} \end{array}$$

for assessing cross-frequency (*f*_*X*_ ≠ *f*_*Y*_) and iso-frequency (*f*_*X*_ = *f*_*Y*_) coupling between signals, where *m*/*n* is the simple whole number ratio of *f*_*X*_/*f*_*Y*_ (e.g., if *f*_*X*_ = 8, *f*_*Y*_ = 16 then *m* = 1, *n* = 2) and

$$\begin{array}{l} S_{XY}(f_{X},f_{Y})=<X^{n}(f_{X}){\left(Y^{m}(f_{Y})\right)}^{*}>,\\ \;S_{X}^{n}(f_{X})=<X^{n}(f_{X}){\left(X^{n}(f_{X})\right)}^{*}>\; \end{array}$$

where < · > represents the averaging over epochs and ${\text{X}}^{\text{n}}=\underset{n}{\underset{︸}{X\left(f_{x}\right)\cdot X\left(f_{x}\right)\cdot \ldots \cdot X\left(f_{x}\right)}}.$

The NMC reflects the strength of iso- or cross-frequency coupling between signals. When *f*_*X*_ = *f*_*Y*_, we have *m* = *n* = 1, then the NMC is equivalent to the classical (linear) coherence for iso-frequency coupling (Yang et al., 2016a). When *f*_*X*_ ≠ *f*_*Y*_, then the NMC indicates the non-linear coupling between signals across different frequency components (i.e., cross-frequency coupling) (Yang et al., 2015). Thus, the n:m mapping can generate harmonic (*m* = 1) and subharmonic coupling (*m* > 1) between the input and the output in the frequency domain (Yang et al., 2016b). As a generalized coherence method, the NMC is a metric indicating cross-frequency coherence between signals, which is different from other cross-frequency coupling methods such as the phase-amplitude coupling (De Hemptinne et al., 2013) reflecting how a low-frequency phase modulates a high-frequency amplitude.

According to Cauchy-Schwarz-inequality, we have:

$$\begin{array}{l} |\langle X^{n}\left(f_{X}\right){\left(Y^{m}\left(f_{Y}\right)\right)}^{*}\rangle |\leq {\left(\langle {\left|X^{n}\left(f_{X}\right)\right|}^{2}\rangle \right)}^{1/2}{\left(\langle {\left|Y^{m}\left(f_{Y}\right)\right|}^{2}\rangle \right)}^{1/2} \end{array}$$

Thus, the NMC is bounded by 0 and 1, where 1 indicates that two signals are perfectly coupled at the tested frequency pair (*f*_X_, *f*_Y_). As the NMC values are computed by comparing different frequency pairs between signals, the significant threshold was adapted with a Bonferroni correction to control the type I error (family-wise error rate: 0.05) (Yang et al., 2016b). There are 2,100 frequency pairs that were included for Bonferroni corrections, i.e., 21 frequencies in the input (from 15 to 35 Hz at 1 Hz resolution) × 100 frequencies in the output (from 1 to 100 Hz at 1 Hz resolution). More details of the NMC method is available in Yang et al. (2016b).

Since the supraspinal input had added independent noise for each 1st layer neuron, each coupling analysis was repeated 100 times, each time with a different realization of the independent noise (as described in section Input Signal and Connection Configuration) added to the supraspinal input in the same signal-to-noise ratio as the original input (i.e., ~-7.5 dB). To compare the dominance of cross- vs. iso-frequency coupling, we defined the cross-frequency coupling over iso-frequency coupling index as COI = (CFC–IFC)/(CFC+IFC), where CFC is the sum of all significant cross-frequency coupling values and IFC the sum of all significant iso-frequency coupling. We included only “significant” CFC and IFC values to exclude false positives in the coherence analysis. The range of COI is [−1, 1], where a larger COI indicates a more dominant cross-frequency coupling.

To examine the effect of the number of synaptic/interneuron layers on neural coupling of descending pathways, we computed NMC, IFC, CFC, and COI between the given supraspinal input and the cumulative spike train (CST) output of the simulated motoneuron pool (derived as the sum of individual motoneuron spike trains following Negro and Farina, 2011a). In addition, the IFC, CFC, and COI between the supraspinal input and the CST of each successive interneuron layer was also computed to evaluate how they change across layers. Furthermore, we also examined the combined effect of both mono- and multi-synaptic pathways on neural coupling by varying the weight of the input from either pathway (with the same supraspinal input) to the terminal motoneuron pool.

## Results

### Neural Coupling Between the Supraspinal Input and the Cumulative Output From Motoneuron Pool

**Figure 4:**
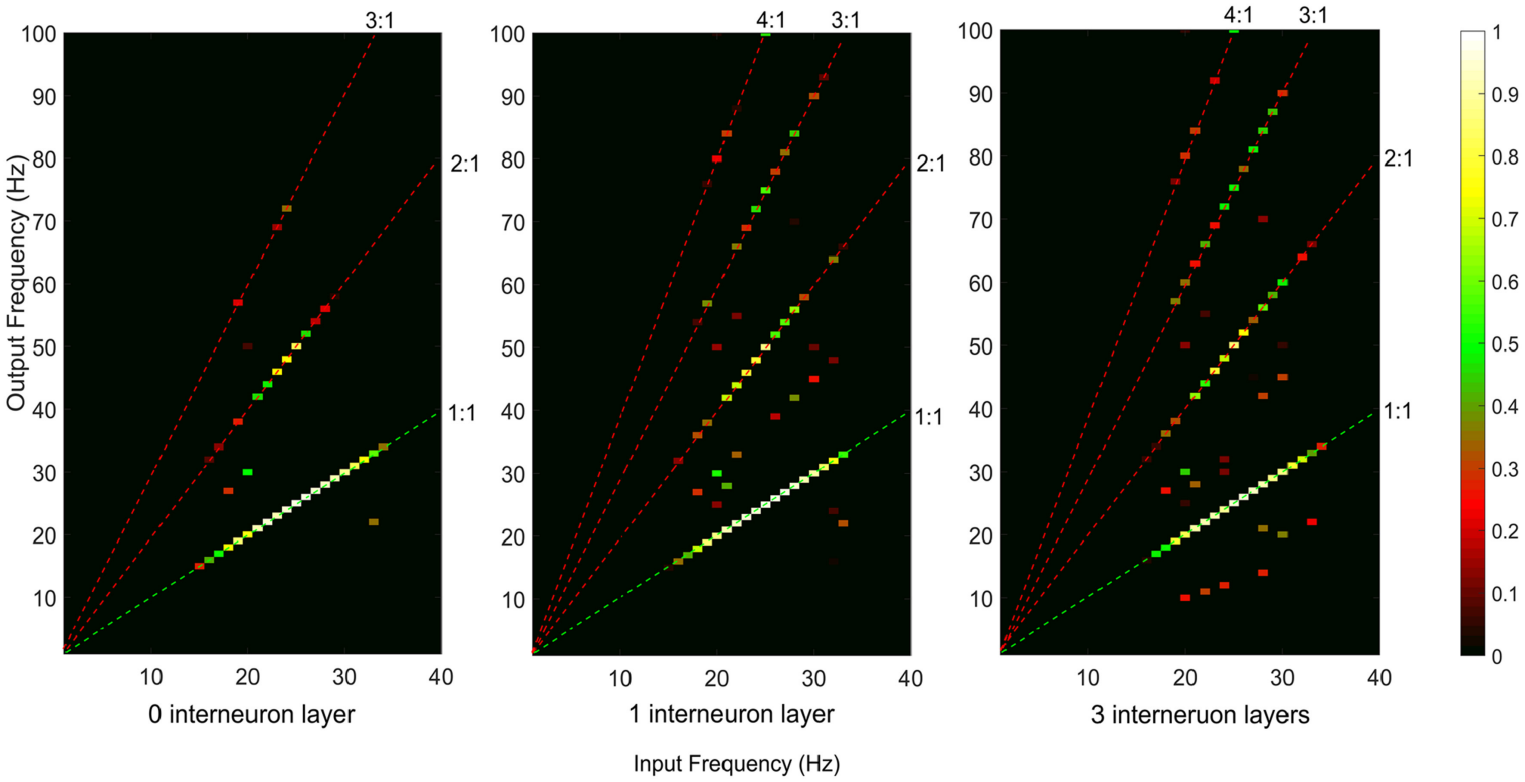
Neural coupling between the supraspinal input and cumulative spike train output of the terminal motoneuron pool for the descending motor pathways with 0, 1, and 3 interneuron layers. The iso-frequency (1:1) coupling is indicated by the green dashed line. The detected cross-frequency coupling including harmonic coupling (i.e., output frequency over input frequency ratio n/m is an integer, e.g., 2:1, 3:1, 4:1, indicated by the red dashed lines) and non-integer n:m coupling (other points in the map).

Both iso-frequency coupling and cross-frequency coupling were detected in the simulated motor pathways (see Figure 4). The detected cross-frequency coupling includes harmonic coupling (i.e., output frequency over input frequency ratio n/m is an integer) and non-integer n:m coupling. This result is in line with previous experimental studies reporting both harmonic and non-integer coupling in the human sensorimotor system (Daffertshofer et al., 2000; Yang et al., 2016a). A higher amount of cross-frequency coupling was observed in the multi-synaptic pathways where there are one or more interneuron layers.

**Figure 5:**
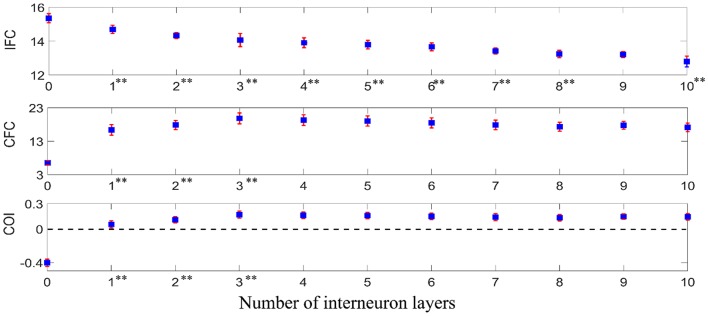
IFC, CFC, and COI between the supraspinal input and the motoneuron pool output in simulated pathways with various layers of interneurons in cascade with a terminal motoneuron pool. The depicted values represent the mean (with ±2 standard deviation as indicated by error bars) calculated from 100 repetitions of the coupling analysis, each being run with a different realization of additive noise to the supraspinal input (as described in section Neural Coupling Analysis). Tukey's test was performed to test for significant decrease in IFC and increase in CFC and COI between the pathways containing *n* − 1 and n (*n* = 1, 2, 3…) interneuron layers. To control the family-wise error rate, we set the threshold *p* = 0.05/*k* (*k* = 10, i.e., number of comparisons). Asterisks in superscript of the n-th layer number indicate a significant change of the results in the n-interneuron layer motor pathway in comparison to (*n* − 1)-interneuron layer motor pathway (**p < 0.05/10).

To examine how iso-frequency coupling and cross-frequency coupling evolved with increasing interneuron layers, we computed the IFC, CFC, and COI between the given supraspinal input and the motoneuron pool output for simulated pathways with various layers (*N* = 0, 1, 2, 3, …) of interneurons in cascade with a terminal motoneuron pool (see Figure 5). Using one-way ANOVA we found that the number of interneuron layers had significant effect on IFC [*F*_(10, 1089)_ = 3613.36, *p* < 0.001], CFC [*F*_(10, 1089)_ = 2934.50, *p* < 0.001] and COI [*F*_(10, 1089)_ = 7108.90, *p* < 0.001]. We used Tukey's honest significant difference (HSD) criterion for *post-hoc* comparisons, with Bonferroni correction to control the type I error. Hence, we adjusted the threshold *p*-value as 0.05/k to control the family-wise error rate to be <0.05, where *k* is the number of *post hoc* comparisons (*k* = 10). We found that the IFC decreased with increasing number of interneuron layers in the pathways (*p* < 0.05/10), while the CFC increased in the pathway with up to three interneuron layers (*p* < 0.05/10). Their combined effect resulted in an initial increase in COI with interneuron layers (*p* < 0.05/10) and a saturation for the pathways with more than three interneuron layers. It was also observed from the COI values that the iso-frequency coupling is only dominant (COI < 0) in the mono-synaptic descending pathway where the supraspinal input directly drives the motoneuron pool without passing through any interneuron layer. Consequently, the cross-frequency coupling became dominant (COI > 0) when there were interneuron layers in the descending pathway.

### Neural Coupling Between the Supraspinal Input and the Output From Successive Neuron Layers

**Figure 6:**
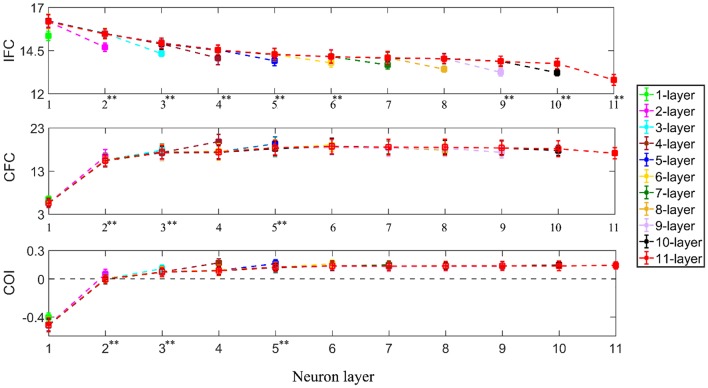
IFC, CFC, and COI between the supraspinal input and cumulative spike train output of successive neuron layers in simulated pathways with various layers of interneurons in cascade with a terminal motoneuron pool. The 1-layer pathway represents the monosynaptic tract. The depicted values represent the mean (with ±2 standard deviation as indicated by error bars) calculated from 100 repetitions of the coupling analysis, each being run with a different realization of additive noise to the supraspinal input (as described in section Neural Coupling Analysis). Tukey's test was performed to test for significant decrease in IFC and increase in CFC and COI between the *n* − 1th and *n*th (*n* = 2, 3, 4…) interneuron layer. To control the family-wise error rate, we set the threshold *p* = 0.05/*k* (*k* = 9, i.e., number of comparisons) Asterisks in superscript of the n-th layer number indicate a significant change of the result in that layer in comparison to that of the previous layer (**p < 0.05/9).

Using one-way ANOVA, we also found that the number of interneuron layers had a significant effect at *p* < 0.05 level on the IFC [*F*_(9, 990)_ = 3235.46, *p* < 0.001], CFC [*F*_(9, 990)_ = 2113.54, *p* < 0.001] and COI [*F*_(9, 990)_ = 5597.45, *p* < 0.001] between the supraspinal input and the output from successive interneuron layers. Using Tukey's HSD criterion with Bonferroni correction (*k* = 9), the IFC was observed to decrease across successive interneuron layers (*p* < 0.05/9) while the CFC and COI increased (*p* < 0.05/9) up to the fifth and sixth layer, respectively (see Figure 6). Additionally, in multi-synaptic pathways, IFC dropped more at the terminal motoneuron layer of the n-layer pathway in comparison to that of the terminal interneuron layer of the *n* + 1 layer pathway (for *n* = 1–11, unpaired *t*-test, *p* < 0.001 in all cases).

### Combined Effect of Mono-Synaptic and Multi-Synaptic Pathways

**Figure 7:**
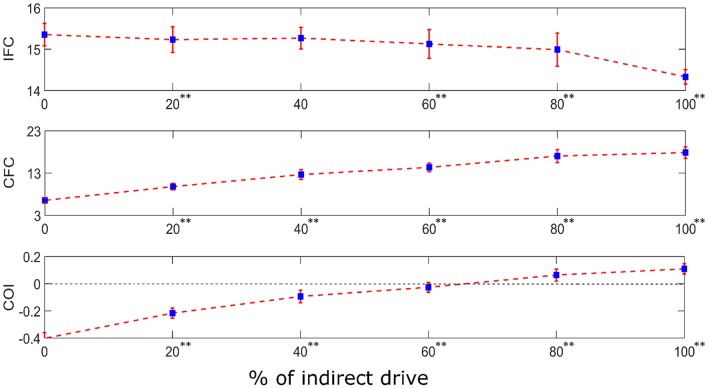
IFC, CFC, and COI between the supraspinal input and cumulative spike train output of the terminal motoneuron pool for the simulated dual input system. The dual input is comprised of (i) a direct supraspinal drive (resembling the monosynaptic corticospinal tract) and (ii) an indirect drive from the same supraspinal input after being passed through two layers of interneurons (resembling a multi-synaptic descending pathway). The relative proportion (in terms of signal power) of the indirect drive in the composite input was systematically varied from 0 to 100% in steps of 20% increments. The depicted values represent the mean (with ±2 standard deviation as indicated by error bars) calculated from 100 repetitions of the coupling analysis, each being run with a different realization of additive noise to the supraspinal input (as described in section Neural Coupling Analysis). Tukey's test was performed to test for significant decrease in IFC and increase in CFC and COI between the successive steps of increase in indirect drive. To control the family-wise error rate, we set the threshold *p* = 0.05/*k* (*k* = 5, i.e., number of comparisons). Asterisks in superscript of the percentage values denote significant change (**p < 0.05/5).

In reality, the motor system contains both the mono-synaptic corticospinal tract and multi-synaptic indirect motor pathways. Hence, to examine the combined effect of both types of descending pathways in a motor system, we performed simulations in a system having dual input to the terminal motoneuron pool. The dual input is comprised of (i) a direct supraspinal drive (resembling the monosynaptic corticospinal tract) and (ii) an indirect drive from the same supraspinal input after being passed through two layers of interneurons (resembling a multi-synaptic descending pathway). The relative weights of these two drives were systematically varied to examine their effects on the neural coupling between the supraspinal input and the motoneuron pool output. Using one-way ANOVA, we found that the proportion of direct vs. indirect drive has a significant effect on the IFC [*F*_(5, 594)_ = 640.63, *p* < 0.001], CFC [*F*_(5, 594)_ = 5552.81, *p* < 0.001] and COI [*F*_(5, 594)_ = 7748.80, *p* < 0.001]. Using Tukey's HSD criterion with Bonferroni correction (*k* = 5), IFC was found to reduce with increased indirect drive (*p* < 0.05/5), while the CFC increased (*p* < 0.05/5). Their combined effect resulted in the progressive increase of COI (*p* < 0.05/5) (see Figure 7).

## Discussion

This study investigated neural coupling in descending motor pathways using computer simulations. We simulated the pathways as various layers of interneurons in a cascade with a terminal motoneuron pool, using Hodgkin–Huxley neuron models, to examine the effect of the number of synapses or interneuron layers on the expression of cross-frequency coupling, as well as its ratio over iso-frequency coupling.

Most studies investigating neural coupling in the descending pathways mainly focus on the mono-synaptic corticospinal tract using iso-frequency coupling measures such as cortico-muscular coherence (Mima and Hallett, 1999; Salenius and Hari, 2003; Negro and Farina, 2011b; van Wijk et al., 2012). In this simulation study, we examined both iso- and cross-frequency coupling in the mono-synaptic descending pathway. Our results confirmed the dominance of iso-frequency coupling (as indicated by COI < 0) in the mono-synaptic pathway, though cross-frequency coupling is also present (see Figure 4). This result is in line with our previous experimental work using the NMC to assess cross- and iso-frequency coupling between brain and muscle signals during a low-effort (1 Nm) isotonic wrist flexion in healthy young participants, showing that the motor task using the mono-synaptic corticospinal tract mainly generates iso-frequency coupling (Yang et al., 2016a).

Using linear coherence measure alone, a previous modeling study indicated that the recruitment of multi-synaptic indirect motor pathways can reduce the iso-frequency coupling between the supraspinal input and motoneuron pool output (Negro and Farina, 2011a). Consistent with the previous study, we found that the IFC decreases in the multi-synaptic pathways: the more interneuron layers in the pathway, the smaller IFC between the supraspinal input and the motoneuron pool output. However, we also found that the CFC initially increases in the multi-synaptic pathways and is then followed by a saturation after passing a few neuron layers. The combined effect of changes in IFC and CFC leads to the dominance of cross-frequency coupling (as shown by COI > 0) in the multi-synaptic pathways.

The mechanism underlying the changes in IFC and CFC could be associated with the information distortion that occurs across neuron layers leading to decorrelation of the supraspinal input and the motoneuron pool output (Negro and Farina, 2011a). Such distortion is likely caused by the modulation of inter-spike intervals when the motor command is passing through multiple synaptic layers (Koch and Segev, 2000; Markram, 2003; Yang et al., 2018). This modulation could be attributed to (1) heterogeneous recruitment thresholds and spike after-hyperpolarizations of the individual neurons which results in different firing rates for the same steady-state drive (Powers and Binder, 2001; Heckman and Enoka, 2012; Yang et al., 2018), and (2) the consequent interplay between the time-varying input (as shown Figure 2, which is added over the steady-state drive) and different firing rates (as shown in Figure 3) of the neurons over the entire pool (Thompson et al., 2018). Thus, besides the input frequecies, the neurons also generate responses at other frequencies, which contain the components that are cross-frequency coupled with the input signal, as well as a certain amount of noise that is not phase-locked to the suprapinal input. Not only the cross-frequency coupled components but also the noise can be cumulatively enhanced when the signal is passing from one layer to the next. After passing a few neuron layers, the reduced signal-to-noise ratio then leads to the saturation of CFC.

In the multi-synaptic pathways, a sharp decrease of IFC was found at the terminal motoneuron pool in comparison to the last interneuron layer. This is likely caused by different neuronal processing properties of the simulated interneurons and motoneurons. The motoneurons modeled in this study had an active dendrite with a persistent inward current as well as calcium dependent potassium currents in both the soma and the dendrite compartments (Heckman et al., 2008). In contrast, the interneurons were modeled without such conductances and had a single compartment only. These differences may have given rise to lower IFC (and higher COI) in motoneuron outputs due to their effects on the neurocomputational properties. Indeed, this study opens a broad new area for exploring the origin of different types of neural coupling at the single neuron level and a detailed analysis of the role of individual ionic conductances on IFC and CFC can be the scope of future studies.

The proposed COI measure reflects the dominance of iso-frequency coupling vs. cross-frequency coupling. Interestingly, the iso-frequency coupling is only dominant in the mono-synaptic pathway, while the cross-frequency coupling is dominant in multi-synaptic pathways. After a unilateral brain injury, damage to the mono-synaptic corticospinal tract can increase the reliance on multi-synaptic indirect motor descending pathways (e.g., cortico-reticulospinal tracts for upper limbs) (Owen et al., 2017; McPherson et al., 2018a; Karbasforoushan et al., 2019). The simulated “dual-drive” model mimicks this pathological condition by varing the ratio of multi-synaptic drive vs. mono-synpatic drive. Our results show that the increased input from the indirect drive leads to a more dominant cross-frequency coupling as reflected by an increased value of COI over the increased percentage of the indirect motor drive. This result is in line with our pilot work on eight participants with hemiparetic stroke. The COI between the brain and muscle signal increases when participants with a unilateral stroke progressively lift the weight of their paretic arm (Yang et al., 2019), thereby enhancing the recruitment of indirect motor pathways to compensate for the loss of corticofugal (i.e., corticospinal and corticobulbar) projections from the lesioned hemisphere (McPherson et al., 2018a).

Thus, the COI can be used as a quantitative measure to indicate the relative usage of multi-synaptic indirect motor pathways vs. mono-synaptic direct corticospinal tract. This measure could have a significant impact on future neuro-pathophysiological studies on individuals with an unilateral brain injury, since recent studies have indicated that motor impairments after a unilateral brain injury could be associated with an increased reliance on multi-synaptic indirect motor pathways following a lesion-induced loss of direct corticospinal projections (Owen et al., 2017; McPherson et al., 2018a; Karbasforoushan et al., 2019). Therefore, a measure that quantitatively determines the usage of indirect motor pathways over direct corticospinal drive could be crucial (1) for evaluating motor recovery following unilateral brain injuries, and (2) for determining the effect of targeted therapeutic interventions (Ellis et al., 2018; McPherson et al., 2018b) that aim to reduce the maladaptive reliance on indirect motor pathways after a hemiparetic stroke. In the future, we will examine both cross-frequency and iso-frequency coupling, as well as the COI, between the brain and muscle signals to characterize the relative ratio of the recruitment of indirect vs. direct motor pathways following unilateral brain injuries, such as hemiparetic stroke and unilateral celebral palsy.

## Limitations

We acknowledged that there are a few limitations of the current study. First, the interneuron model has only a basic set of ionic conductance since the details of the ionic conductances of spinal and reticular interneurons are yet to be explored. However, such reductionist interneuron models have been used in other simulation studies as well, and this simplification is not expected to change the overall results of this study (Maltenfort et al., 1998; Cisi and Kohn, 2008; Williams and Baker, 2009; Negro and Farina, 2011a). Second, there is no existing literature detailing the connection pattern of individual interneuron layers in multi-synaptic descending pathways. However, the probabilistic model used in this simulation has been previously demonstrated to capture global connectivity properties in motor descending pathways well (Humplik and Tkačik, 2017). Thirdly, the number of neurons in each layer of multi-synaptic descending tracts have not yet been experimentally determined. However, compound EPSP recording on motoneurons from spinal interneurons has shown the number of inputs to be ~ 100 (Lüscher et al., 1983). Hence, it is reasonable to assume that the number of descending inputs on the motoneurons from each tract should be in this order. The motoneuron pool size can vary from muscle to muscle (Karpati et al., 2001), in a range from around 100 (e.g., first dorsal interosseous in humans) (Buchthal and Schmalbruch, 1980) to around 800 (e.g., biceps brachii in humans) (Feinstein et al., 1955). Thus, a size of 100 units for a motoneuron pool falls in the lowest part of the range. In this study, the size of 100 units per neuron layer was adopted also for uniformity and computational convenience. Finally, we did not consider synaptic and transmission delays in this work. The overall delay in a motor pathway may be determined based on the onset latency of Transcranial Magnetic Stimulation (TMS) induced Motor Evoked Potential (MEP) in the targeted muscle (Schwerin et al., 2011). However, the latency of MEP can be affected by coil orientation: difference may exist between direct vs. trans-synaptic activation of the pyramidal cells and the measurement of MEP responses in proximal vs. distal muscles. In short, it is still hard to get a “precise” assessment of the delay in a motor pathway. Meanwhile, there is no available experimental data for the delays in each neuron layer that we can included in this simulation work. Moreover, the time delay only has the effect on the relative phase between signals. This will not result in additional resonance components with new frequencies. Thus, the time delay issue will not affect our current results and overall conclusion of this paper.

## Data Availability Statement

The datasets generated for this study are available on request to the corresponding author.

## Author Contributions

NS conducted the simulation and data analysis under the supervision of YY, JD, and CH. NS and YY drafted and wrote the manuscript. JD and CH commented and revised the manuscript.

### Conflict of Interest

The authors declare that the research was conducted in the absence of any commercial or financial relationships that could be construed as a potential conflict of interest.

## Appendix

### Equilibrium Potentials

*E*_*Na*_ = 55*mV*; *E*_*K*_ = −80*mV*; *E*_*Ca*_ = 80*mV*;

*E*_*L*(*S*)_ = −65*mV* ±0.3; *E*_*L*(*D*)_ = −65 ±0.15*mV* (motoneurons*)

*Motoneuron equilibrium potentials were assigned from uniform random distributions with mean ± S.D as given above to obtain firing rates in the range of 5–30 spikes/s as described in section Simulations.

**Interneuron equilibrium potentials were set as described in section Simulations.

### Neuron Paramteres

Motoneurons:

$g_{Na\left(S\right)}=120\;mS\;cm^{-2};\;g_{NaP\left(S\right)}=0.1\;mS\;cm^{-2};\;g_{K\left(S\right)}=100\;mS\;cm^{-2};\;$

$g_{CaN\left(S\right)}=14\;mS\;cm^{-2};\;g_{K,Ca\left(S\right)}=5\;mS\;cm^{-2};\;g_{L\left(S\right)}=0.51\;mS\;cm^{-2};\;$

$g_{CaN\left(D\right)}=0.3\;mS\;cm^{-2};\;g_{CaL\left(D\right)}=0.33\;mS\;cm^{-2};\;g_{NaP\left(D\right)}=0.1\;mS\;cm^{-2};\;$

$g_{K,Ca\left(D\right)}=1.1\;mS\;cm^{-2};\;g_{L\left(D\right)}=0.51\;mS\;cm^{-2};\;g_{c}=0.1\;mS\;cm^{-2};\;$

$p=0.1;\;f=0.01;\;\alpha =0.0009\;mol\;C^{-1}\;\mu m^{-1};\;k_{Ca}=2ms^{-1};\;K_{d}=0.2\mu M\;$

Interneurons:

$g_{Na}=120\;mS\;cm^{-2};\;g_{K}=100\;mS\;cm^{-2};\;g_{L}=0.51\;mS\;cm^{-2};\;$

Synapses:

$E_{synE}=-10mV;\;g_{synE}=0.01mS\;cm^{-2};\;\tau_{synE}=5ms;$

$E_{synE}=-10mV;\;g_{synE}=0.0075mS\;cm^{-2};\;\tau_{synE}=5ms;\;$

**TABLE A1:** Steady-state activation and inactivation variables and time constants for voltage-dependent ionic channels (Rybak et al., 2006).

Ionic channels	*m*_∞_(*V*), *V is in mV* *h*_∞_(*V*), *V is in mV*	τ_*m*_(*V*), *ms* τ_*h*_(*V*), *ms*
*Na*^+^	$m_{\infty \text{Na}}=\;\frac{1}{1+\;e^{-\frac{\left(V+35\right)}{7.8}}}\;$ $h_{\infty Na}=\;\frac{1}{1+\;e^{\frac{\left(V+55\right)}{7}}}\;$	τ_*mNa*_ = 0 $\tau_{hNa}=\;\frac{30}{e^{\frac{\left(V+50\right)}{15}}+\;e^{-\frac{\left(V+50\right)}{16}}}$
*NaP*^+^	$m_{\infty NaP}=\;\frac{1}{1+\;e^{-\frac{\left(V+47.1\right)}{3.1}}}$ $h_{\infty NaP}=\;\frac{1}{1+\;e^{-\frac{\left(V+59\right)}{8}}}$	τ_*mNaP*_ = 0 $\tau_{hNaP}=\;\frac{1,200}{\text{cosh}\left(\frac{\left(V+59\right)}{16}\right)}$
*K*^+^	$m_{\infty K\;}=\;\frac{1}{1+\;e^{-\frac{\left(V+28\right)}{15}}}\;$ *h*_*K*_ = 1	$\tau_{mK}=\;\frac{7}{e^{\frac{\left(V+40\right)}{40}}+\;e^{-\frac{\left(V+40\right)}{50}}}$
*CaN*^2+^	$m_{\infty CaN\;}=\;\frac{1}{1+\;e^{-\frac{\left(V+30\right)}{5}}}\;$ $h_{\infty CaN\;}=\;\frac{1}{1+\;e^{-\frac{\left(V+45\right)}{5}}}$	τ_*mCaN*_ = 4 τ_*hCaN*_ = 40
*CaL*^2+^	$m_{\infty CaL\;}=\;\frac{1}{1+\;e^{-\frac{\left(V+40\right)}{7}}}$ *h*_*CaL*_ = 1	τ_*mCaL*_ = 4;
